# Physcomitrium *LATERAL SUPPRESSOR* genes promote formative cell divisions to produce germ cell lineages in both male and female gametangia

**DOI:** 10.1111/nph.20372

**Published:** 2024-12-31

**Authors:** Yuta Horiuchi, Naoyuki Umakawa, Rina Otani, Yosuke Tamada, Ken Kosetsu, Yuji Hiwatashi, Rena Wakisaka, Saiko Yoshida, Takashi Murata, Mitsuyasu Hasebe, Masaki Ishikawa, Rumiko Kofuji

**Affiliations:** ^1^ National Institute for Basic Biology Okazaki 444‐8585 Japan; ^2^ Basic Biology Program The Graduate University for Advanced Studies (SOKENDAI) Okazaki 444‐8585 Japan; ^3^ Graduate School of Natural Science and Technology Kanazawa University Kanazawa 920‐1192 Ishikawa Japan; ^4^ School of Biological Science and Technology Kanazawa University Kanazawa 920‐1192 Ishikawa Japan; ^5^ School of Engineering Utsunomiya University Utsunomiya 321‐8585 Japan; ^6^ School of Food Industrial Sciences Miyagi University Sendai 982‐0215 Japan; ^7^ Department of Applied Chemistry and Bioscience Kanagawa Institute of Technology Atsugi 243‐0292 Japan

**Keywords:** antheridium, archegonium, formative cell division, gametangium, GRAS, *Physcomitrium*

## Abstract

- The evolution of green plants from aquatic to terrestrial environments is thought to have been facilitated by the acquisition of gametangia, specialized multicellular organs housing gametes. Antheridia and archegonia, responsible for producing and protecting sperm and egg cells, undergo formative cell divisions to produce a cell to differentiate into germ cell lineages and the other cell to give rise to surrounding structures. However, the genes governing this process remain unidentified.
- We isolated genes expressed during gametangia development from previously established gene‐trap lines of *Physcomitrium patens* and characterized their function during gametangia formation.
- We identified *P. patens LATERAL SUPPRESSOR 1* (*PpLAS1*) from the gene‐trap library, encoding a GRAS transcription factor. The double‐deletion mutant with its paralog *PpLAS2* failed to form inner cells in both gametangia. PpLASs are expressed in cells undergoing formative cell division, and introducing PpLAS1 into the double‐deletion mutant successfully rescued the phenotype.
- These findings underscore the pivotal role of PpLASs in regulating formative cell divisions, ensuring the separation of reproductive cell lineages from surrounding cells in antheridia and archegonia. Furthermore, they suggest a link between PpLASs and the evolutionary origin of male and female gametangia in the common ancestor of land plants.

## Introduction

Land plant lineage diverged from aquatic green algal ancestor *c*. 490 million years ago at the latest (Donoghue *et al*., 2021; Bowman, 2022). The subsequent transition to land was marked by the evolution of specific morphological traits adapted for terrestrial life (Kenrick & Crane, 1997; Doyle, 2017; de Vries & Archibald, 2018; Bowman, 2022). One of novel traits in this transition was the emergence of gametangia: multicellular reproductive organs consisting of the egg‐bearing archegonium and the sperm‐bearing antheridium. Both structures protect and nourish differentiating germ cells, with the archegonium additionally supporting the growing embryos. These facilitate adaptation to terrestrial habitats. While gametangia are retained in extant non‐seed plants as seen in the ancestral land plants (Remy *et al*., 1993; Taylor *et al*., 2005), they are reduced to several or no cells in seed plants, in which gametophytes are epiphytic to sporophytes (Gifford & Foster, 1989; Crum, 2001). Despite variations in the development and morphology of gametangia among land plant lineages, formative cell divisions play a central role, generating asymmetric daughter cells that spatially organize into inner and outer cell layers (Renzaglia, 1978; Gifford & Foster, 1989; Crum, 2001; Kofuji & Hasebe, 2014; Kohchi *et al*., 2021). These layers subsequently differentiate, with inner cells giving rise to germ cell lineages and outer cells forming the surrounding structure.

In bryophytes, several genes have been implicated in gametangium and gamete formation (Koi *et al*., 2016; Rövekamp *et al*., 2016; Koshimizu *et al*., 2018; Yamaoka *et al*., 2018; Hisanaga *et al*., 2019; Sanchez‐Vera *et al*., 2022; Bao *et al*., 2024). However, the molecular mechanisms governing the precise orientation of cell division, crucial for the correct formation of inner and outer cell layers in these reproductive organs, remain elusive. The GRAS transcription factor gene family is proposed to have been acquired at the common ancestor between Zygnematophyceae and land plants through horizontal gene transfer from soil bacteria, subsequently diverging into several subfamilies (Zhang *et al*., 2012; Nishiyama *et al*., 2018; Cheng *et al*., 2019). In flowering plants, *SHORT‐ROOT* (*SHR*) and *SCARECROW* (*SCR*) genes within this family regulate formative periclinal cell divisions in the roots (Di Laurenzio *et al*., 1996; Helariutta *et al*., 2000). In the moss *Physcomitrium patens* (*P. patens*), two *SHR* and one *SCR* genes along with another GRAS family gene *LATERAL SUPPRESSOR* (*LAS*), cooperatively participate in the formation of leaf veins, including water‐conducting systems, by regulating cell division orientation (Ge *et al*., 2022; Ishikawa *et al*., 2023). This suggests that the acquisition of GRAS genes contributed to establishing and elaborating genetic regulatory networks controlling formative cell divisions during land plant evolution.

This study aimed to elucidate the roles of two *P. patens LAS* genes, *PpLAS1* and *PpLAS2* (Ge *et al*., 2022; Ishikawa *et al*., 2023), in shaping the inner and outer cell layers during antheridium and archegonium development. Both *PpLAS* genes were expressed in apical stem cells and the adjacent daughter cells in both reproductive organs. Remarkably, the deletion of both *PpLAS1* and *PpLAS2* disrupted the formation of inner and outer cells in these organs, underscoring the crucial role of *PpLAS* genes in directing formative cell divisions producing the inner and outer cells during the development of both male and female gametangia.

## Materials and Methods

### Plant materials and culture conditions

The Cove‐NIBB strain of *Physcomitrium patens* (Hedw.) Mitt. was used as the wild‐type (WT) strain (Nishiyama *et al*., 2000). The ∆pplas1, ∆pplas2, ∆pplas1∆pplas2, ∆ppshr1∆ppshr2, PpLAS1‐mClover3, PpLAS2‐mClover3, PpSHR1‐mCitrine, PpSHR2‐mCitrine, PpSHR1‐Citrine∆pplas1∆pplas2, and PpSHR2‐Citrine∆pplas1∆pplas2, nPpLAS1pro:XVE>PpLAS1‐Citrine∆pplas1∆pplas2, PGX8:NGG, and PGX8:PpLAS1‐Citrine lines were reported in Ishikawa *et al*. (2023). Protonemata cultivated on a BCDAT agar plate (Nishiyama *et al*., 2000) were transplanted into sterile peat pellets (Jiffy‐7) and cultured for 1 month at 25°C under long‐day conditions (16 h : 8 h, light : dark). To induce gametangia, the peat pellets were moved to 15°C under short‐day conditions (8 h : 16 h, light : dark).

To examine the effects of cytokinin on gametangia development, gametophores of WT and ∆pplas1∆pplas2 mutant plants were cultured on BCD agar media (Nishiyama *et al*., 2000) for 5 wk under 25°C under long‐day (16 h : 8 h, light : dark) conditions. Subsequently, the gametophores were transferred to solid BCDAT media containing 100 μg l^−1^ kinetin at 15°C under short‐day conditions (8 h : 16 h, light : dark) for 3 wk to induce antheridia and archegonia.

### Thermal asymmetric interlaced (tail)‐PCR

Tail‐PCR (Liu *et al*., 1995; Liu & Whittier, 1995) was employed to isolate genomic fragments flanking inserted *uidA* genes in the gene‐trap lines (Hiwatashi *et al*., 2001). Primers used for Tail‐PCR are listed in Supporting Information Table S1. Genomic DNA was extracted from protonemata of the Gt9 line (Kofuji *et al*., 2009), and the putative integration regions were amplified through three consecutive rounds of PCR. Three types of initial PCR products were obtained with three primer pairs combining *uidA*‐specific primer GUS‐R4 and one of the degenerate primers A1, A2, or A3. Subsequently, each initial PCR product was diluted 50‐fold and used to generate second PCR products with *uidA‐*specific primer GUS‐R3, which is upstream of GUS‐R4, and the same degenerate primer used in the initial PCR. The products from the second PCR, diluted 100‐fold, underwent a third amplification with *uidA*‐specific primer GUSseq‐ and the same degenerate primer used in the initial and the second PCRs. The resulting DNA junction fragments were subcloned into a pGEM‐T vector (Promega) and sequenced.

### Microscopy and cell segmentation

Bright‐field images of the WT and deletion mutants were acquired under the fluorescence microscopes BX51 or BX63 (Olympus, Tokyo, Japan). For cell segmentation of gametangia, gametophores with gametangia were fixed in a fixation solution (4% (w/v) paraformaldehyde), 100 μg ml^−1^ Calcofluor white M2R (Fluorescent Brightener 28; Sigma‐Aldrich) in phosphate‐buffered saline (PBS) for 3 d at 4°C, washed twice for 5 min each in PBS, and immersed in ClearSee (Kurihara *et al*., 2015) solutions (10% (w/v) xylitol powder, 12.5% (w/v) sodium deoxycholate, 25% (w/v) urea in water) at room temperature for 2 d. The ClearSee solution was replaced once with a fresh solution. The SP8 confocal microscope (LEICA, Wetzlar, Germany) equipped with a 405 nm laser, a white light laser, and hybrid detectors (HyD) was used for the imaging with ×40 1.10‐NA water‐immersion objective lenses. Fluorescence from calcofluor white was recorded at 0.25 μm Z‐intervals. Segmentation of individual cells was performed using MorphoGraphX (de Reuille *et al*., 2015) (https://morphographx.org) based on the fluorescence signal of Calcofluor white. To observe fluorescent signals from PpLAS‐mClover3 fusion proteins, gametophores with gametangia of PpLAS1‐mClover3 and PpLAS2‐mClover3 plants were fixed in 4% (w/v) paraformaldehyde in PBS and stained with Calcofluor white in PBS. Fluorescent images were recorded at 0.25 μm Z‐intervals. The 3D stacks of images were loaded into Fiji (https://imagej.net/software/fiji/) to select optical sections for use, and affine transformations were applied to obtain longitudinal sections.

### Induction of PpLAS1‐Citrine

To induce the PpLAS1‐Citrine fusion protein during gametangium development in nPpLAS1pro:XVE>PpLAS1‐Citrine∆pplas1∆pplas2 line, gametophores were cultured on BCD agar media (Nishiyama *et al*., 2000) for 5 wk under 25°C under long‐day (16 h : 8 h, light : dark) conditions. Subsequently, gametophores of nPpLAS1pro:XVE>PpLAS1‐Citrine∆pplas1∆pplas2 were transferred into liquid BCD media containing 1 μM β‐estradiol at 15°C under short‐day conditions (8 h : 16 h, light : dark) for 2 wk to observe antheridia. As archegonia form after the initiation of antheridia, 1 μM β‐estradiol was added after 7 d of culture in the liquid media at 15°C under short‐day conditions, and induced archegonia were observed after 12 d.

In the GX8:NGG and GX8:PpLAS1‐Citrine lines, gametophores were cultured at 15°C under short‐day conditions for 13 d, transferred to solid BCDAT medium containing 1 μM β‐estradiol, and then incubated for an additional 8 d. The gametophore leaves were removed from the gametophores to expose the antheridia and archegonia, which were then fixed with a fixation solution for observation described above.

## Results

### LATERAL SUPPRESSOR expresses during gametangia development

To identify genes expressed in both antheridia and archegonia, we conducted screening using gene‐trap and enhancer‐trap lines of *P. patens* (Hiwatashi *et al*., 2001; Kofuji *et al*., 2009). In the previous study (Kofuji *et al*., 2009), we found 30 gene‐trap lines with β‐glucuronidase (GUS) activity in both antheridia and archegonia from 5400 gene‐trap lines (Hiwatashi *et al*., 2001). We isolated a DNA fragment adjacent to the GUS‐tag sequence using tail‐PCR from one of the 30 lines and found that the sequence was identical to a partial sequence of the *PpLAS1* gene (Fig. S1; Ge *et al*., 2022; Ishikawa *et al*., 2023). *PpLAS1* regulates the direction of cell division during leaf development by regulating the expression of the *PpSHR* genes. This prompted an investigation into the potential roles of *PpLAS* genes in regulating cell division orientation during gametangium development.

### PpLASs function in antheridium and archegonium development

**Fig. 1:**
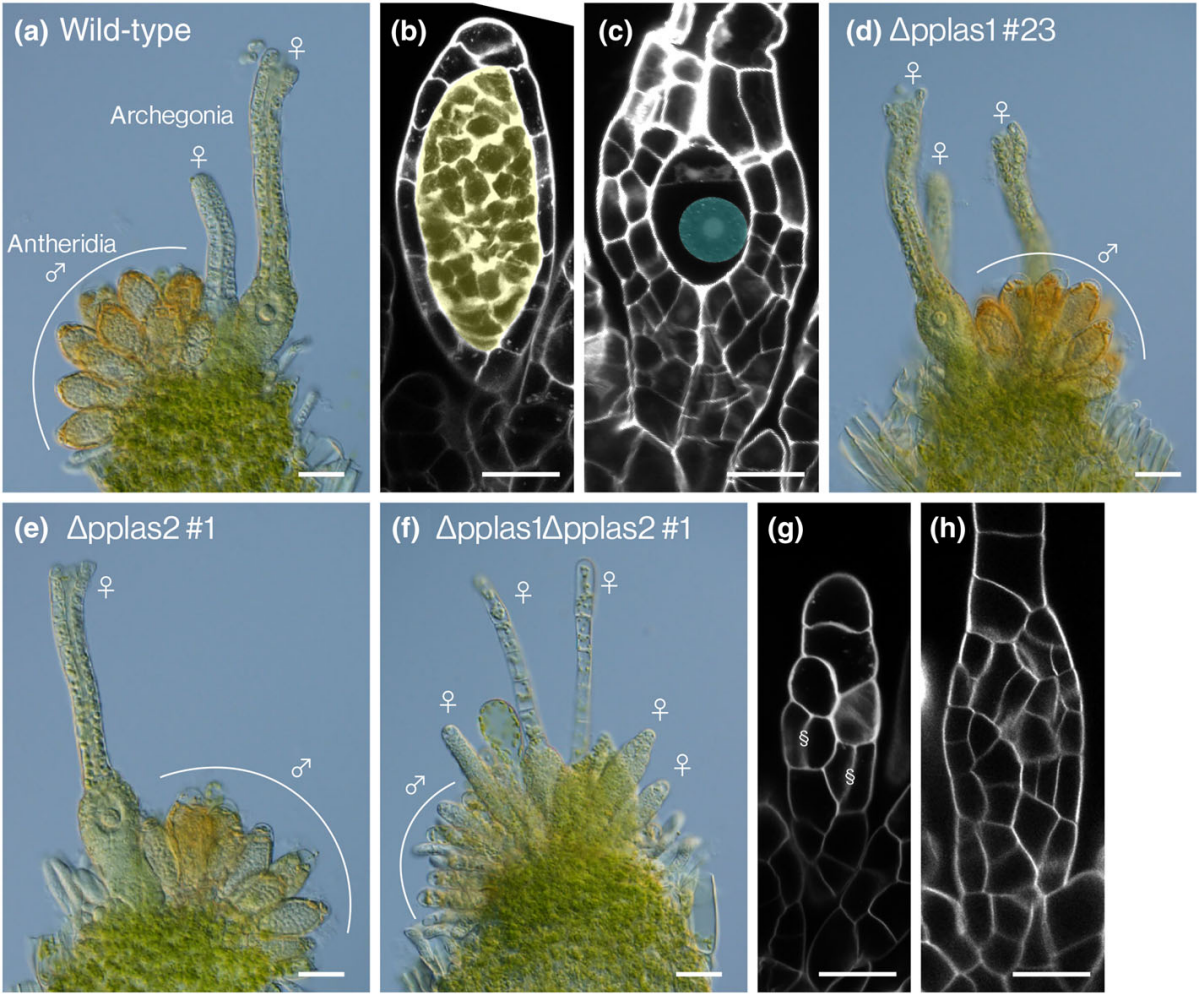
Involvement of *Physcomitrium patens LATERAL SUPPRESSOR* (*PpLAS*) genes in antheridium and archegonium development of *Physcomitrium patens*. (a–c) Representative antheridia and archegonia at the gametophore apex in the wild‐type (WT) (a) plants. ‘♂’ and ‘♀’ indicate the antheridia and the archegonium, respectively. Optical sections of an antheridium (b) and an archegonium (c) in WT, showing spermatogenous cells (yellow) and an egg cell (cyan). (d–h) Representative antheridia and archegonia at the gametophore apex in the ∆pplas1#23 (d), ∆pplas2#1 (e), and ∆pplas1∆pplas2#1 (f). Optical sections of an antheridium (g) and an archegonium (h) in ∆pplas1∆pplas2#1, indicating the absence of spermatogenous cells and egg cells. Longitudinal‐anticlinal division planes in (g) are indicated as ‘§’ (see text). Bars: (a, d, e, f) 50 μm; (b, c, g, h) 20 μm. The same set of data was obtained from independently generated transgenic lines, and the phenotypes were not distinguished from those in this figure (Supporting Information Fig. S2).

To investigate the functions of *PpLAS* genes in gametangium development, we analyzed single and double‐deletion mutant lines for *PpLAS1* and its paralogous *PpLAS2* (Ishikawa *et al*., 2023). ∆pplas1, ∆pplas2, and ∆pplas1∆pplas2 lines previously reported (Ishikawa *et al*., 2023) were cultivated under the gametangia‐inductive conditions (Hohe *et al*., 2002). In the WT, antheridia containing sperm and archegonia housing the egg cell are formed in discrete clusters after 1 month (Fig. 1a–c). The clustered antheridia and archegonia in each ∆pplas1 and ∆pplas2 single‐deletion mutants were indistinguishable from those in the WT (Figs 1d,e, S2a,b). On the other hand, antheridia and archegonia of ∆pplas1∆pplas2 double‐deletion mutant lines were malformed (Figs 1f–h, S2c), and sperm and egg cells failed to form (Figs 1g,h, S2d,e), indicating that *PpLAS*s redundantly function in forming proper gametangia and facilitating sperm and egg differentiation.

### *PpLAS*s govern formative cell divisions to produce a reproductive cell lineage in antheridia

**Fig. 2:**
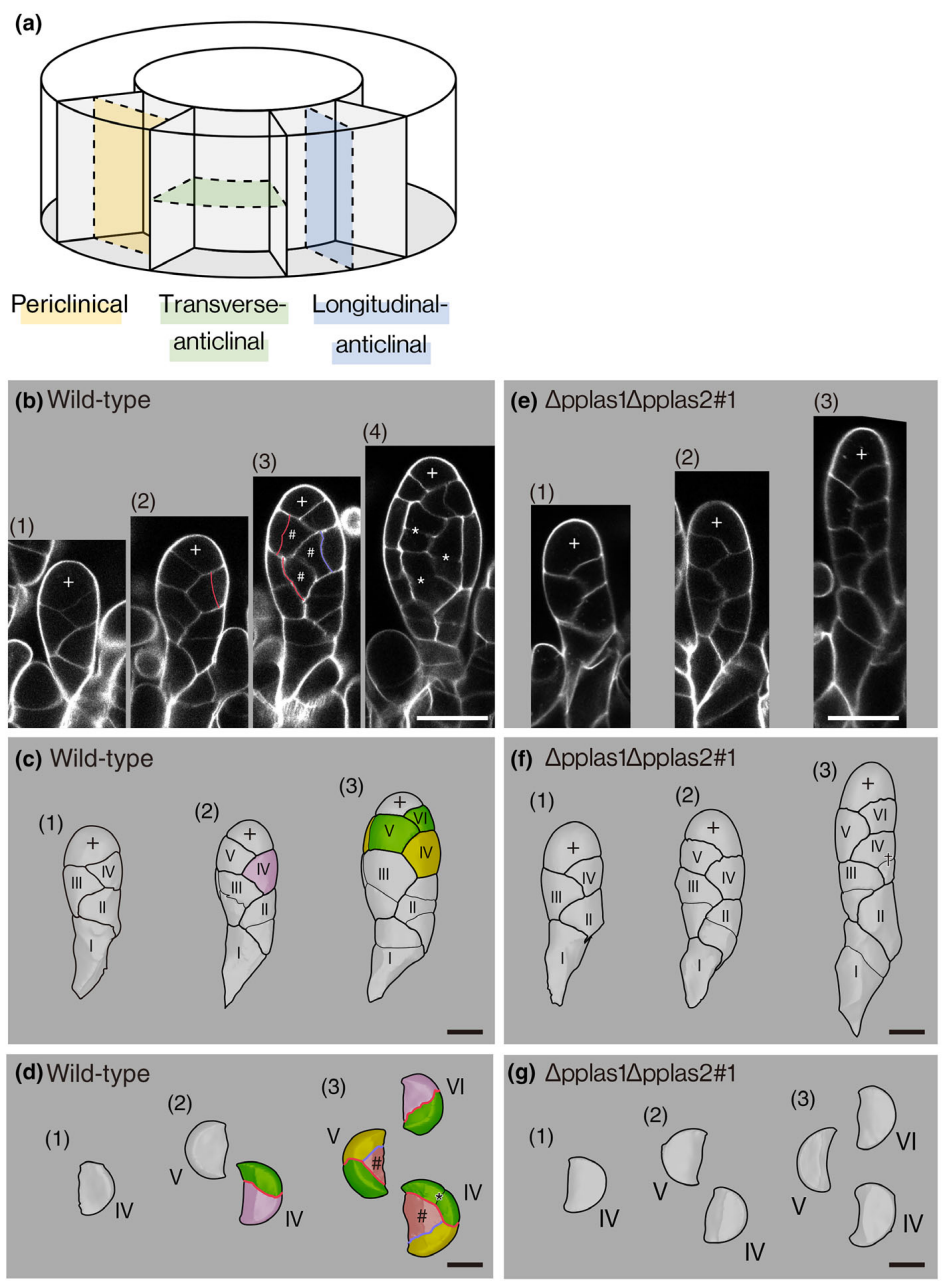
Regulation of cell division orientation by *Physcomitrium patens LATERAL SUPPRESSOR* (*PpLAS*s) in antheridium development of *Physcomitrium patens*. (a) A schematic of cell division orientation. (b–g) Developmental stages of antheridia in wild‐type (WT) (b–d) and ∆pplas1∆pplas2#1 deletion mutant (e–g). (b, e) Representative optical longitudinal sections of developmental stages are shown in the same scale. Bars, 20 μm. Cell walls are visualized with calcofluor white. Antheridium apical stem cells are denoted by ‘+’. Red and purple lines indicate longitudinal‐anticlinal and periclinal cell division planes, respectively, that produce inner and outer cell layers. Inner cells are marked with ‘#’. Transverse division planes in the inner cells are marked with asterisks. Three‐dimensional (3D) reconstructions of antheridia of developmental stage (1) to (3) of (b) and (e) are shown in (c), (d), (f), and (g) using MorphoGraphX (de Reuille *et al*., 2015). ‘Segments’ produced from the antheridium apical stem cell are numbered in Roman numerals from older to younger ones. (c, f) Surface side views of antheridia. ‘†’ in (f) (3) indicates a transverse‐anticlinal division plane. (d, g) Top views of each ‘segment’. Different cell lineages are indicated by green, pink, yellow, and orange colors, as explained in the text. Inner cells that become generative cells are marked with ‘#’. A series of optical sections for each gametophore apex in WT (*n* = 31) and ∆pplas1∆pplas2#1 (*n* = 26) were subjected to 3D projection analysis. Each 3D image contained multiple antheridia. A total of 71 WT and 116 mutant antheridia were analyzed for morphological characteristics. This analysis confirmed consistent cell division patterns across the observed developmental stages in each genotype. Bars, 10 μm.

**Fig. 3:**
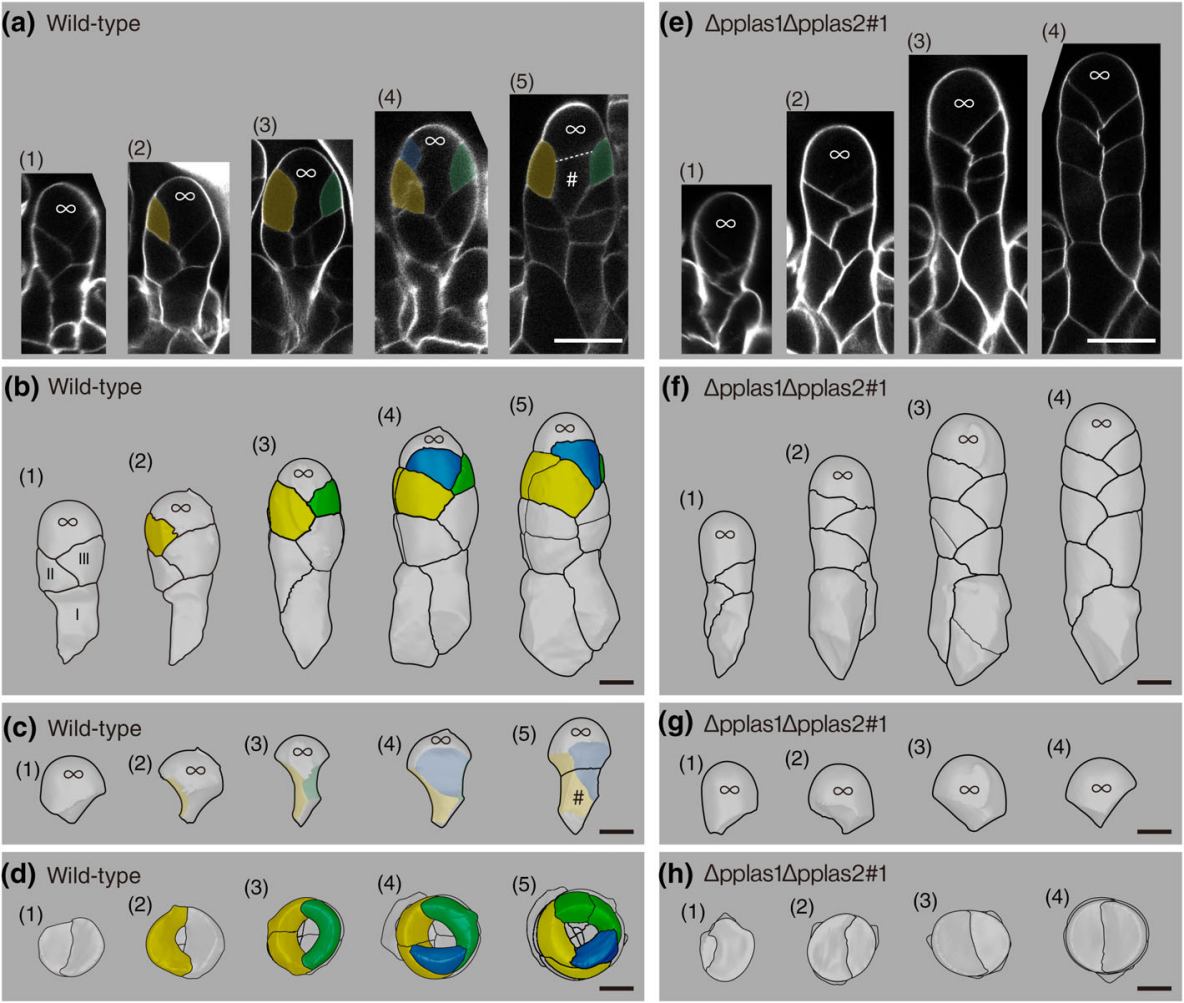
Regulation of cell division orientation by *Physcomitrium patens LATERAL SUPPRESSOR* (*PpLAS*s) in archegonium development of *Physcomitrium patens*. Developmental stages of archegonia in wild‐type (WT) (a–d) and ∆pplas1∆pplas2#1 deletion mutant (e–h). (a, e) Representative optical longitudinal sections depicting developmental stages at the same scale. Bars, 20 μm. Cell walls are visualized with calcofluor white. The archegonium apical stem cell is denoted by ‘∞’. The fourth, fifth, and sixth segments, and seventh cell division planes, generating the inner cell marked by ‘#’ in WT, are marked by yellow, green, blue, and a white dotted line, respectively. (b–d, f–h) Three‐dimensional reconstructions of segmented cells using MorphoGraphX (de Reuille *et al*., 2015) at different developmental stages. The numbers of developmental stages correspond to those in (a) and (e). (b, c, f, g) Surface side views of archegonia (b, f) and archegonium apical stem cells (c, g). (c) The fourth, fifth, and sixth cell division planes, leading to the vertically elongated archegonium apical stem cell, are marked by yellow, green, and blue (c). (d, h) Top views of archegonia from which the archegonium apical stem cell is removed. (b, d) The fourth, fifth, and sixth segments are colored in yellow, green, and blue. A series of optical sections were acquired from 15 WT and 6 ∆pplas1∆pplas2#1 archegonia. A total of 15 WT and six mutant images were selected for three‐dimensional projection analysis. This analysis confirmed consistent cell division patterns across all developmental stages in each genotype. Representative images from each stage are presented here. Bars, 10 μm.

To investigate the cellular basis of gametangium defects in ∆pplas1∆pplas2 mutants. We investigated cell division patterns during antheridium and archegonium development with a series of confocal optical sections (Figs 2b,e, 3a,e), which were three‐dimensionally constructed (Figs 2c,d,f,g, 3b–d,f–h). In the WT, the antheridium apical stem cell (designated with ‘+’ in Fig. 2b,e) distichously produced wedge‐shaped cells called ‘segments’ in the proximal direction (Kofuji *et al*., 2018). The segments are numbered in Roman numerals from older to younger ones in Fig. 2c,d. The fourth and later segments asymmetrically divided to the longitudinal‐anticlinal direction (division planes: red lines in Fig. 2b,d; ‘§’ in Fig. 1g; blue plane in Fig. 2a), yielding larger and smaller daughter cells (pink and green cells, respectively, in IV segment in Fig. 2d(2) and VI segment in Fig. 2d(3)). The larger daughter cells successively divided periclinally (purple line in Fig. 2b(3) and purple lines in V and IV segments of Fig. 2d(3); yellow plane in Fig. 2a), resulting in the formation of inner and outer cells. Inner cells are indicated with ‘#’ in Fig. 2b(3) and Fig. 2d(3). The inner cell further divided to produce spermatogenous cells (yellow cells in Fig. 1b), while the outer cells (yellow and green cells in Fig. 2c,d) contributed to the formation of antheridium jacket cells. Subsequently, both inner and outer cells underwent transverse‐anticlinal division (e.g. cell septums with asterisks in Fig. 2b(4); green plane in Fig. 2a) and longitudinal‐anticlinal divisions (a division plane of IV segment with an asterisk in Fig. 2d(3)) to enlarge the antheridium without changing their cell fates. In Δpplas1Δpplas2 mutant lines, the antheridium apical stem cell produced segments as observed in the WT (Fig. 2f,g), although the volume of the apical stem cell is larger than that of WT (904 ± 89 μm^3^ (*n* = 8) in WT and 1577 ± 138 μm^3^ (*n* = 7) in the mutant; *P* = 0.00104 by the two‐tailed Student's *t*‐test). Furthermore, two consecutive longitudinal‐anticlinal cell divisions that lead to the formation of the inner cell in the WT were not observed in the mutant (Fig. 2e–g). The fourth and later segments in the mutant underwent longitudinal‐anticlinal divisions (division planes with ‘§’ in Fig. 1g) and transverse‐anticlinal (a division plane with ‘†’ in Fig. 2f(3)) divisions but failed to generate inner and outer cells. These observations underscore the role of *PpLAS1* and *PpLAS2* in governing cell divisions that contribute to the formation of the inner reproductive and outer vegetative cell lineages during antheridium development.

### *PpLAS*s manage formative cell divisions to produce a reproductive cell lineage in archegonia

We subsequently compared the development of archegonia between WT and the double mutant (Fig. 3). In the WT, the archegonium apical stem cell usually produced three distichous segments in the proximal direction (Fig. 3a(1) and segments numbered in Roman numerals in Fig. 3b(1)). While second and third division planes of the archegonium apical stem cell are mostly flat, the fourth, fifth, and sixth planes are concaved toward the central axis, resulting concaved cells (yellow, green, and blue cells in Fig. 3d). The second to the fifth division planes were oriented at an *c*. 180‐degree angle to the preceding division plane, whereas the sixth division plane tended to be less angled (Fig. 3d). The fourth to sixth divisions produced the archegonium apical stem cell adopting a vertically elongated shape, with its proximal part facing and being surrounded by lateral three cells (Fig. 3a–c(4)). The seventh cell division of the archegonium apical stem cell was transverse (a white dotted line in Fig. 3a(5)), and the daughter cell (the cell marked with ‘#’ in Fig. 3a(5),c(5)) became an inner cell referred to as a central cell, surrounded by the fourth, fifth, and sixth segments and the archegonium apical stem cell. The inner cell successively divided to form an egg cell, while the fourth, fifth, and sixth cells divided to form archegonium venter tissue (Lal & Bhandari, 1968).

In the double‐deletion mutant, the fourth and subsequent division planes of the archegonium apical stem cell were not concave to the central axis, leading to the absence of inner cell formation (Fig. 3e–h). Distichously arranged cells divided, forming venter‐like jacket tissue without an egg cell (Fig. 1h). These observations indicate that *PpLAS*s play a crucial role in regulating cell division orientations, contributing to the formation of the gamete and jacket cell lineages in both male and female gametangia.

### PpLASs localize to cells undergoing formative cell divisions in reproductive organ development

**Fig. 4:**
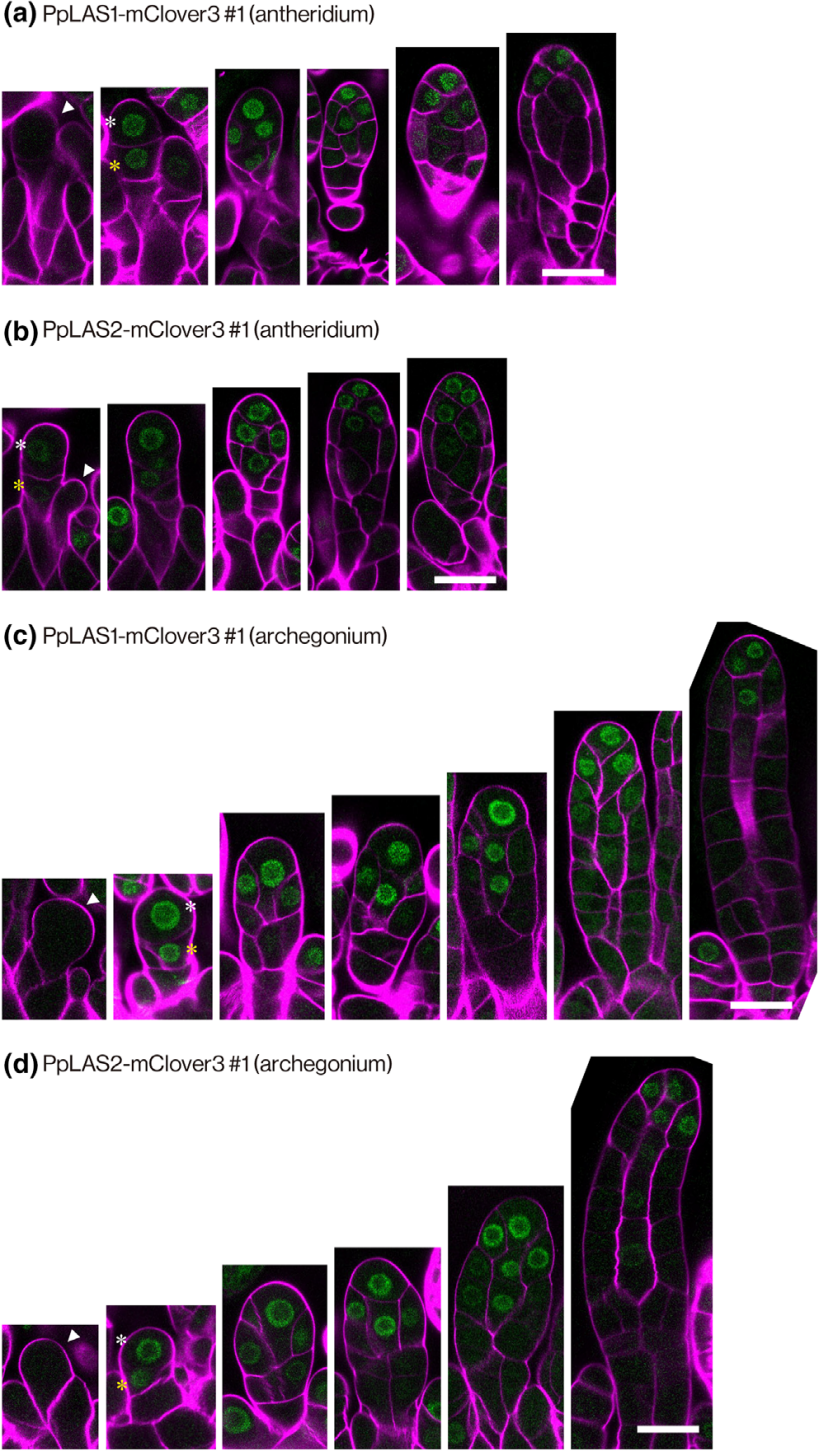
Accumulation of *Physcomitrium patens* LATERAL SUPPRESSOR 1 (PpLAS1) and *Physcomitrium patens* LATERAL SUPPRESSOR 2 (PpLAS2) fusion proteins during gametangium development of *Physcomitrium patens*. (a–d) Representative spatial accumulation patterns of PpLAS1 (a, c) and PpLAS2 (b, d) fused to mClover3 fluorescent protein (green) during antheridium (a, b) and archegonium (c, d) development. Cell walls of antheridia and archegonia in PpLAS1‐mClover3#1 and PpLAS2‐mClover3#1 lines were visualized with Calcofluor white (magenta). Arrowheads indicate the antheridium apical stem cell (a, b) and the archegonium apical stem cell (c, d) before the first cell division. White and yellow asterisks indicate apical stem cells and segment cells, respectively. Bars, 20 μm. The same set of data was obtained from independently generated transgenic lines and the phenotypes were not distinguished from those in this figure (Supporting Information Fig. S3).

We next investigated the localization of PpLAS1 and PpLAS2 proteins during antheridia and archegonia development using PpLAS1‐mClover3 and PpLAS2‐mClover3 plants, in which gene coding the fluorescent protein mClover3 (Bajar *et al*., 2016) was inserted just before the stop codon of *PpLAS1* and *PpLAS2* loci, respectively (Ishikawa *et al*., 2023). Neither PpLAS1‐mClover3 nor PpLAS2‐mClover3 was detected in protruding antheridium and archegonium apical stem cells (arrowheads in Figs 4a–d, S3a–d) before the first division. After the first cell division, PpLAS1‐mClover3 and PpLAS2‐mClover3 signals were detected in apical stem cells (white asterisks) and segment cells (yellow asterisks) in both gametangia (Figs 4a–d, S3a–d). In the antheridium, both signals were similarly detected in the antheridium apical stem cell and the segment cells that underwent the longitudinal‐anticlinal and subsequent periclinal cell divisions to form the inner cell (Figs 4a,b, S3a,b). In the archegonium, both signals were continuously detected in the archegonium apical stem cell, including the period during which the inner cell was formed (Figs 4c,d, S3c,d). Together, PpLAS1 and PpLAS2 fusion proteins were co‐expressed in cells responsible for formative cell divisions to produce reproductive cells during the development of male and female gametangia.

### Distinct gene regulatory networks of *PpLAS*s in gametangia compared to leaf vein development

**Fig. 5:**
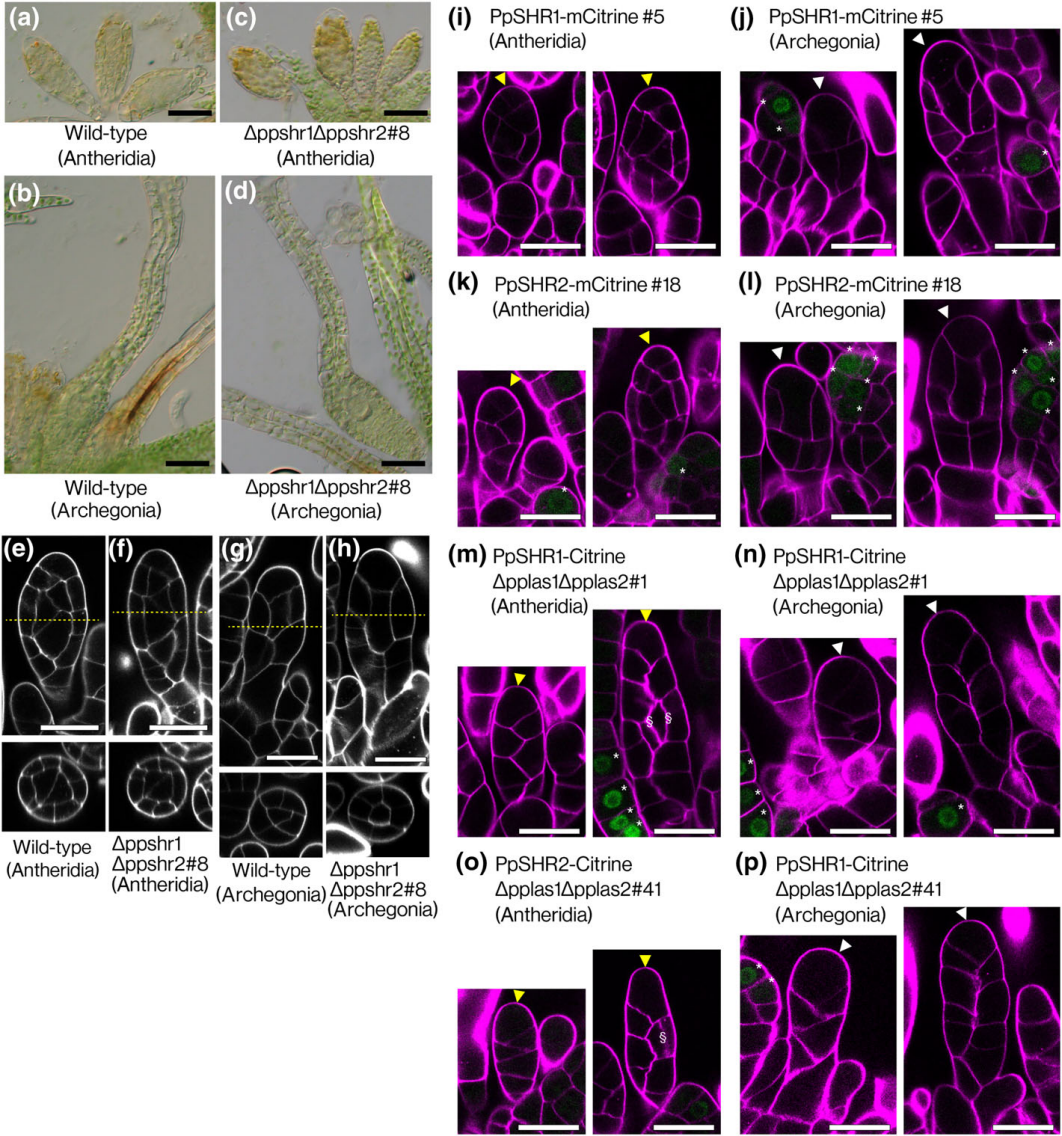
*Physcomitrium patens* SHORT‐ROOTs (PpSHRs) are dispensable for gametangium development of *Physcomitrium patens*. (a–d) Representative antheridia (a, c) and archegonia (b, d) of wild‐type (WT) (a, b) and ∆ppshr1∆ppshr2#8 (c, d) plants. Antheridia and archegonia in ∆ppshr1∆ppshr2#8 plant were indistinguishable from those in the WT. This phenotype was consistent with observations from an independently generated Δppshr1Δppshr2#7 line (Supporting Information Fig. S4). (e–h) Optical sections of an antheridium (e, f) and an archegonium (g, h) in WT (e, g) and in ∆ppshr1∆ppshr2#8 (f, h). Optical transverse sections along the yellow dotted lines (upper) are shown in the lower panels. (i–l) Representative spatial accumulation patterns of PpSHR1 (i, j) and PpSHR2 (k, l) fused to mCitrine fluorescent protein (green) during antheridium (i, k) and archegonium (j, l) development in PpSHR1‐mCitrine#5 and PpSHR2‐mCitrine#18 plants. (m–p) Representative spatial accumulation patterns of PpSHR1 (m, n) and PpSHR2 (o, p) fused to Citrine fluorescent protein (green) during antheridium (m, o) and archegonium (n, p) development in PpSHR1‐Citrine∆pplas1∆pplas2#1 and PpSHR2‐Citrine∆pplas1∆pplas2#41 plants. Cell walls of antheridia (i, k, m, o) and archegonia (j, l, n, p) were visualized with Calcofluor white (magenta). Yellow and white arrowheads indicate developing antheridia (i, k, m, o) and archegonia (j, l, n, p), respectively. Note that mCitrine and Citrine signals (asterisks) were detected in leaf primordia but not in any cells within gametangia. Longitudinal‐anticlinal division planes in (m, o) are indicated as ‘§’. Bars: (a–d) 50 μm; (e–p) 20 μm.

*PpLAS*s negatively regulate *SHORT‐ROOT* (*SHR*) during leaf development (Ishikawa *et al*., 2023). To investigate whether this genetic regulatory network also operates in gametangium development, we examined the ∆ppshr1∆ppshr2 deletion mutant lines. We found that the mutants formed normal antheridia and archegonia indistinguishable from those in the WT (Figs 5a–d, S4a). Importantly, the cell division patterns that generate the inner and outer cell layers in the antheridia and archegonia of Δppshr1Δppshr2 mutants were indistinguishable from those of WT plant (Fig. 5e–h).

We also examined the localization of PpSHR1 and PpSHR2 proteins during antheridium and archegonium development using PpSHR1‐mCitrine and PpSHR2‐mCitrine plants (Ishikawa *et al*., 2023). Neither PpSHR1‐mCitrine nor PpSHR2‐mCitrine was detected before and after inner cell formation in developing gametangia, while both proteins were present in leaf primordia (Fig. 5i–l). Next, we investigated the effect of the *PpLAS1* and *PpLAS2* gene deletion on PpSHR expression during gametangium development using the PpSHR1‐Citrine∆pplas1∆pplas2 and PpSHR2‐Citrine∆pplas1∆pplas2 lines (Ishikawa *et al*., 2023). No signal of PpSHR1‐Citrine or PpSHR2‐Citrine was observed in these gametangia (Fig. 5m–p). These results indicate that PpSHRs are not implicated in gametangia development and that PpLASs do not regulate the expression of PpSHR1 and PpSHR2 during this process. This suggests that PpLASs function within a distinct gene regulatory network to generate inner and outer cells in gametangia, differing from the PpLAS‐PpSHR gene regulatory network in leaf vein development.

### Precise spatiotemporal expression of PpLAS is indispensable for formative cell division

**Fig. 6:**
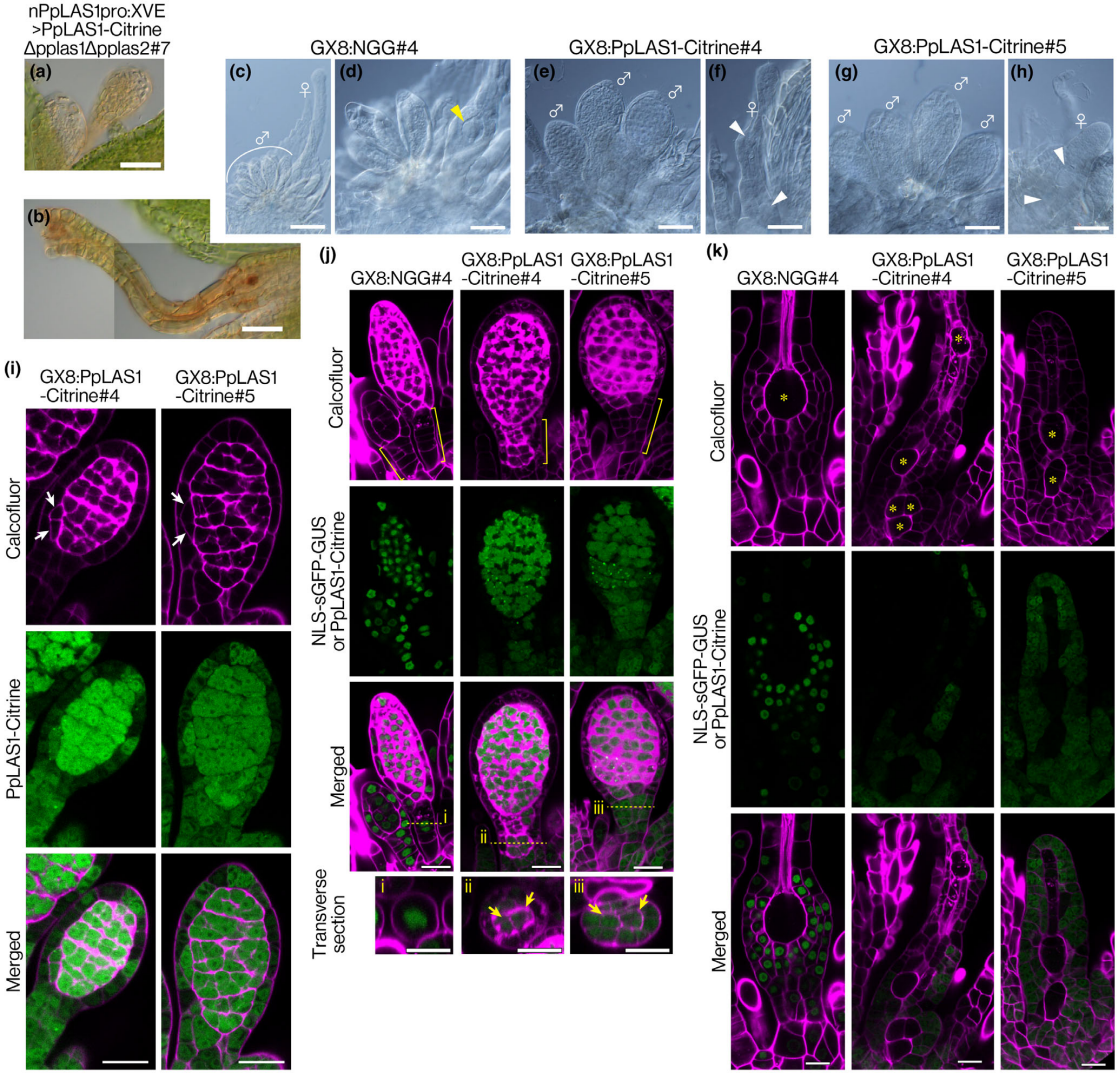
Induction of PpLAS1‐Citrine in antheridia and archegonia of *Physcomitrium patens*. (a, b) Two antheridia (a) and an archegonium (b) of nPpLAS1pro:XVE>PpLAS1‐Citrine∆pplas1∆pplas2#7 cultivated for 2 wk in the presence of 1 μM β‐estradiol. Two images are aligned to flame the entire archegonium in (b). Note that the gametangia formed after induction were not distinguished from those in the wild‐type (WT). The phenotypes obtained from independently generated transgenic line, nPpLAS1pro:XVE>PpLAS1‐Citrine∆pplas1∆pplas2#1, were not distinguished from those in this figure (Supporting Information Fig. S4). (c, d) Representative antheridia (♂) and archegonia (♀) of the GX8:NGG#4 line ectopically expressing a nuclear‐localized sGFP‐GUS (NLS‐sGFP‐GUS) fusion protein for 1 wk in the presence of 1 μM β‐estradiol. A magnified view of antheridia and an archegonium in (c) is shown in (d). Yellow arrowhead indicates an egg cell within an archegonium. (e–h) Representative antheridia (♂) and archegonia (♀) of the GX8:PpLAS1‐Citrine#4 and #5 lines ectopically expressing PpLAS1‐Citrine fusion protein for 1 wk in the presence of 1 μM β‐estradiol. Note that the formation of distinct egg cells was impaired in archegonia ectopically expressing PpLAS1‐Citrine (f, h) compared to those expressing NLS‐sGFP‐GUS protein (d). White arrow heads indicate cavities containing a cell resembling the ventral cell. (i–k) Representative optical sections of antheridia (i, j) and archegonia (k) of GX8:NGG#4, GX8:PpLAS1‐Citrine#4 and GX8:PpLAS1‐Citrine#5 cultivated in the presence of 1 μM β‐estradiol. The gametophores of these lines were transferred to gametogenesis‐inducing conditions. On the specified days, the gametophores were moved to BCDAT medium containing β‐estradiol: 8 d after transfer (i) and 13 d after transfer (j, k), followed by an additional 8 d of culture. Cell walls of antheridia and archegonia in all lines were visualized with Calcofluor white (magenta). NLS‐sGFP‐GUS and PpLAS1‐Citrine fluorescence is shown in green. Merged images are also displayed. White arrows in (i) indicate ectopic periclinal cell divisions in jacket cells. Yellow brackets in (j) indicate the antheridia stalks. Optical transverse sections, corresponding to the yellow dotted lines in the longitudinal sections (i, ii, and iii), are shown below (j), with yellow arrows indicating extra inner cell formation in antheridia stalks. Asterisks in (k) indicate cavities. Note that the optical sections of the GX8:PpLAS1‐Citrine#4 and #5 lines in (k) are derived from the archegonia shown in (f) and (h), respectively. Bars: (a, b, d–h) 50 μm, (c) 100 μm, (i–k) 20 μm.

We then investigated the effect of PpLAS1 overexpression using nPpLAS1pro:XVE>PpLAS1‐Citrine∆pplas1∆pplas2 lines. In these lines, *PpLAS1‐Citrine*, controlled by the native *PpLAS1* promoter activity, is introduced into the ∆pplas1∆pplas2 double‐deletion mutant (Ishikawa *et al*., 2023). The induction of *PpLAS1* in leaves changes the *PpSHR* expression level and leads to an increase in the number of leaf vein cells (Ishikawa *et al*., 2023). Inducible lines preceded the development of both gametangia beyond the stage of inner and outer cell formation and produced normal antheridia and archegonia (Figs 6a,b, S4b,c), indicating that *PpLAS1* regulates the formative cell divisions.

Next, we induced PpLAS1‐Citrine during gametangium development in a WT background using GX8:PpLAS1‐Citrine lines (Ishikawa *et al*., 2023). Although induction with this system is not uniform (Kubo *et al*., 2013), we observed substantial increase in antheridia size in GX8:PpLAS1‐Citrine#4 and #5 lines, with 16 of 19 and 34 of 38 antheridia, respectively, being larger than WT antheridia (Fig. 6c–e, g–j). In the enlarged antheridia, the number of androgonial cells appeared to increase, although precise counting was not possible due to ectopic PpLAS1‐Citrine localization in the cytosol, which obscures cell boundaries (Fig. 6i). Occasionally, jacket cells divided to produce an additional inner androgonial cell (nine of 17 in the #4 line and seven of 10 antheridia in the #5 line; Fig. 6i). Additional inner cells also formed in the stalks (16 of 19 in the #4 line and 24 of 38 in the #5 line, yellow brackets and optical transverse sections shown in (i), (ii), and (iii); Fig. 6j). In the archegonia of the GX8:PpLAS1‐Citrine lines, we observed extra cavities containing a cell resembling the ventral cell (three of eight in the #4 line and four of nine archegonia in the #5 line; Fig. 6f,h,k). These results align with the hypothesized function of *PpLAS1* in promoting formative cell divisions to generate reproductive cells. Together, these findings indicate that precise spatiotemporal expression of *PpLAS1* is necessary for proper gametangium development.

Given that exogenous cytokinin partially rescues the lack of midrib formation in Δpplas1Δpplas2 mutants by promoting cell division (Ge *et al*., 2022), we treated the gametangia of wild‐type and Δpplas1Δpplas2 mutant plants with kinetin under gametangia‐inducing conditions. However, exogenous kinetin did not rescue antheridium formation in Δpplas1Δpplas2 mutants (Fig. S5). Moreover, archegonium development was inhibited in both wild‐type and Δpplas1Δpplas2 mutant plants in the presence of kinetin (Fig. S5).

## Discussion

Gametangia play a crucial role in protecting and nursing gametes, enabling the successful adaptation of green plants to terrestrial environments with harsh conditions (Kenrick & Crane, 1997; Doyle, 2017; Bowman, 2022). This study showed the essential role of two GRAS transcription factors, PpLAS1 and PpLAS2, in orchestrating formative cell divisions that give rise to the inner cell lineage, forming gametes, and the outer cell lineage, constituting the enveloping vegetative cell layers in both antheridia and archegonia (Figs 2, 3). Despite distinct patterns of cell divisions between the two structures, the regulatory influence of PpLASs remains integral. In the antheridium, successive longitudinal‐anticlinal and periclinal divisions of segment cells, daughter cells of the segment cell, generate inner and outer cells (Fig. 2). The inner cells subsequently divide to produce spermatogenous cells (Lal & Bhandari, 1968). On the other hand, in the archegonium, three successive oblique divisions of the archegonium apical stem cell result in three lateral segments and an apical stem cell to the center, the latter followed by a transverse division of the apical stem cell to produce an inner cell (Fig. 3). The inner cell, called the central cell, further divides to form the egg cell (Lal & Bhandari, 1968; Kofuji *et al*., 2009). Since PpLASs were expressed in the subsequent stages of archegonium development (Fig. 4c,d), they may function in these processes.

Several genes are involved in gametangium and gamete formation in bryophytes. *BONOBO* (*MpBNB*), which encodes a basic helix–loop–helix transcription factor, is indispensable for initiating the primordial cell of gametangiophores, on which gametangia are formed in the liverwort *Marchantia polymorpha* (Yamaoka *et al*., 2018). Gametangiophores are specific to liverworts and are not formed in mosses including *P. patens*. In deletion mutants of the *P. patens BNB* genes, malformed gametangia with inner cells were produced (Sanchez‐Vera *et al*., 2022), suggesting that *PpBNB* does not play a role in forming the central cell, where PpLASs are functional. Additionally, in deletion mutants of *P. patens MALE STERILITY 1* (*MS1*) and *MALE MEIOCYTE DEATH 1* (*MMD1*), which encode a protein with the Plant Homeo Domain motif, inner cells are formed in both gametangia, indicating that these genes function in the later developmental stages (Landberg *et al*., 2022). Unlike in leaf veins, where *PpLAS*s genetically interact with other GRAS transcription factors (Ishikawa *et al*., 2023), no genes genetically interacting with *PpLAS*s during the formation of inner cells have been reported. Further studies are necessary to understand the genetic regulatory networks involved in the formative cell divisions in gametangia. Histidine kinase CYTOKININ‐INDEPENDENT 1 (CKI1) is critical for the asymmetric cell divisions that specify the female reproductive cells in *M. polymorpha* (Bao *et al*., 2024). On the other hand, *P. patens* lacks its homolog, suggesting that bryophytes acquired distinct regulatory networks to govern periclinal cell divisions in reproductive cell formation between different lineages. Understanding the genes downstream of *PpLAS* will be essential to elucidate the mechanisms through which *PpLAS* influences gametangium development and germ cell specification in *P. patens*. These insights may also provide broader context on the evolutionary origins of plant reproductive structures, revealing how regulatory networks for reproductive cell division diversified among land plants.

Beyond the formation of gametangia, PpLASs also regulate formative cell divisions for the development of inner and outer cells in leaves (Ge *et al*., 2022; Ishikawa *et al*., 2023). Mosses, including *P. patens*, produce water‐conducting cells known as hydroid cells in stems and leaves. In *P. patens*, segment cells derived from the leaf apical stem cell divide longitudinal‐anticlinally or transverse‐anticlinally to form a leaf lamina, with periclinal cell divisions in the medial region generating outer epidermal cells and inner water‐conducting cells, some of which differentiate into hydroids (Ishikawa *et al*., 2023). While formative periclinal cell divisions for water‐conducting cells generally adhere to the geometry rule of dividing along the plane with the minimum surface area (Errera, 1886; Besson & Dumais, 2011; Robinson, 2021), cell proliferation for leaf lamina formation sometimes does not follow this rule, as two *PpSHR* genes redundantly override it (Ishikawa *et al*., 2023). *PpSCR* and *PpLAS* positively and negatively regulate *PpSHR* expression, respectively (Ishikawa *et al*., 2023). On the other hand, in gametangia development, PpLAS did not regulate PpSHR expression, and PpSHR itself was not implicated in the development of these reproductive organs. However, it still remains unknown whether formative cell divisions in gametangia adhere to the geometry rule, because gametangia are covered with young leaves and we could not capture cells right after cytokinesis to employ the minimum surface area simulation (Ishikawa *et al*., 2023). Additionally, it is unclear whether PpLASs may function in promoting cell division to change cell division orientation or cell morphology, leading to the generation of inner cells.

Despite the shared involvement of PpLASs in regulating formative cell divisions in these three organs with pivotal roles for the landing of green plants, the patterns of cell division to form inner and outer cells differ, suggesting the engagement of PpLASs in distinct genetic regulatory networks as putative transcription factors. In flowering plants, *LAS* genes contribute to the formation of axillary meristems, facilitating lateral branching (Schumacher *et al*., 1999; Greb *et al*., 2003; Li *et al*., 2003). Notably, commercial watermelon *LAS* exhibits pleiotropic functions, influencing tendril and flower identities, leaf morphology, and axillary meristem formation, accompanied by changes in associated proteins (Jiang *et al*., 2023). Further investigations into the interacting proteins with PpLASs will provide valuable insights into their multifaceted functions in *P. patens*. It is particularly noteworthy that the horizontal transfer of the GRAS family of transcription factors from soil bacteria to the algal ancestor of land plants facilitated the evolution of water‐conducting tissue, antheridia, and archegonia – three innovative features crucial for survival in terrestrial environments.

## Competing interests

None declared.

## Author contributions

Y Horiuchi, MH, MI and RK conceived and designed the research. Y Horiuchi, NU, RO, YT, KK, Y Hiwatashi, RW, SY, TM, MI and RK performed the experiments. Y Horiuchi, MH, MI and RK wrote the manuscript and incorporated comments from all authors.

## Disclaimer

The New Phytologist Foundation remains neutral with regard to jurisdictional claims in maps and in any institutional affiliations.

## Supporting information


**Fig. S1** Isolation of the *Physcomitrium patens LATERAL SUPPRESSOR 1* gene in *Physcomitrium patens*.
**Fig. S2** Antheridium and archegonium development in ∆pplas1#26, ∆pplas2#16, and ∆pplas1∆pplas2#2 plants of *Physcomitrium patens*.
**Fig. S3** Accumulation of *Physcomitrium patens* LATERAL SUPRESSOR 1 and *P. patens* LATERAL SUPRESSOR 2 fusion proteins during gametangium development in *P. patens*.
**Fig. S4** Formation of gametangia in ∆ppshr1∆ppshr2#7 and nPpLAS1pro:XVE>PpLAS1‐Citrine∆pplas1∆pplas2#1 plants of *Physcomitrium patens*.
**Fig. S5** Effect of cytokinin on antheridia and archegonia development in *Physcomitrium patens*.
**Table S1** Primer sequences used for tail‐PCR.
Please note: Wiley is not responsible for the content or functionality of any Supporting Information supplied by the authors. Any queries (other than missing material) should be directed to the *New Phytologist* Central Office.

## Data Availability

The data that support the findings of this study are available in the article and in Figs S1–S5 and Table S1.
