# Supplementary material for: Physcomitrium LATERAL SUPPRESSOR genes promote formative cell divisions to produce germ cell lineages in both male and female gametangia
Source: New Phytol. 2024 Dec 31;245(5):2004–15. doi: 10.1111/nph.20372 (PMC11798890; doi:10.1111/nph.20372)
Supplement: Supplementary file 1 — Fig. S1 Isolation of the Physcomitrium patens LATERAL SUPPRESSOR 1 gene in Physcomitrium patens. Fig. S2 Antheridium and archegonium development in ∆pplas1#26, ∆pplas2#16, and ∆pplas1∆pplas2#2 plants of Physcomitrium patens. Fig. S3 Accumulation of Physcomitrium patens LATERAL SUPRESSOR 1 and P. patens LATERAL SUPRESSOR 2 fusion proteins during gametangium development in P. patens. Fig. S4 Formation of gametangia in ∆ppshr1∆ppshr2#7 and nPpLAS1pro:XVE>PpLAS1‐Citrine∆pplas1∆pplas2#1 plants of Physcomitrium patens. Fig. S5 Effect of cytokinin on antheridia and archegonia development in Physcomitrium patens. Table S1 Primer sequences used for tail‐PCR. Please note: Wiley is not responsible for the content or functionality of any Supporting Information supplied by the authors. Any queries (other than missing material) should be directed to the New Phytologist Central Office. [file NPH-245-2004-s001.pdf]

New Phytologist **Supporting Information**

Article title: **Physcomitrium *LATERAL SUPPRESSOR* genes promote formative cell divisions to produce germ cell lineages in both male and female gametangia**

Authors: Yuta Horiuchi, Naoyuki Umakawa, Rina Otani, Youske Tamada, Ken Kosetsu, Yuji Hiwatashi, Rena Wakisaka, Saiko Yoshida, Takashi Murata, Mitsuyasu Hasebe, Masaki Ishikawa, Rumiko Kofuji

Article acceptance date: 10 December 2024

The following Supporting Information is available for this article:

Fig. S1. Isolation of the *Physcomitrium patens LATERAL SUPPRESSOR 1* gene in *Physcomitrium patens*.

Fig. S2. Antheridium and archegonium development in Δpplas1#26, Δpplas2#16, and Δpplas1Δpplas2#2 plants of *Physcomitrium patens*.

Fig. S3. Accumulation of *Physcomitrium patens* LATERAL SUPPRESSOR 1 and *P. patens* LATERAL SUPPRESSOR 2 fusion proteins during gametangium development in *Physcomitrium patens*.

Fig. S4. Formation of gametangia in Δppshr1Δppshr2#7 and nPpLAS1pro:XVE>PpLAS1-CitrineΔpplas1Δpplas2#1 plants of *Physcomitrium patens*.

Fig. S5. Effect of cytokinin on antheridia and archegonia development in *Physcomitrium patens*.

Table S1. Primer sequences used for tail-PCR.

New Phytologist

Fig. S1. Isolation of the *PpLAS1* gene in *Physcomitrium patens*.

(**a**) Schematic diagram of the HI-GT gene trap element. The element is a mini-transposon containing 38 bp terminal repeats of the Tn3 transposon (TR) (Hiwatashi et al., 2001). It contains a factor Xa protease cleavage site (XA), a loxP site (loxP), the *Arabidopsis GPA1* intron, the *uidA*-coding region (uidA), the nopaline synthase polyadenylation signal (nos-ter), an NPTII expression cassette (nptII), an ampicillin-resistance gene (amp^{r}), a Tn3 transposon *res* site (res), and three-repeated HA epitopes (3xHA). (**b**) Sequence analysis of the HI-GT insertion site in one of the 30 GUS-positive Lines (See the text). Tail-PCR was performed to identify the genomic region flanking the HI-GT element in the line. The sequence of the DNA fragment is shown, with cyan and black characters representing the partial sequences of PpLAS1 and HI-GT, respectively.

(**a**)

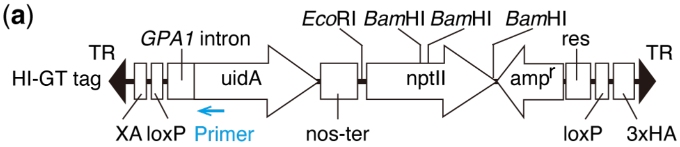

(**b**)

CAAGATGTTA CTTGGATGCC AAGAGGAGAC ACAAAATAGT GGACGATCTT
GCCAACCCAA CAATAAGCAC CCGTCCTCAA CGTCCTCCAC AAGCCGATAA
CCATCGCAAG GGTAATGAAG TCGTAGAAGT AGTCTAGCTT GTGACGTCGC
AAACCTGCTT GTCGGCAGCA ACTGAAACCC AGCTCTCTCG AAGAATCTGC
GCCACTGCTC ACATCGCTGG TGTCTAACTG TGCGCTCCGT CCCATCACAT
GCAACGATGT TGATAATCTC CATTTTATAC CACGTCTGTT CAACTCTGAT
TCGCTCAGGG CTAGTCGGAG GCAACGTCGC ATCCAGGCTG TCGAACAGAG

LAS1 ← → HI-GT tag sequence

TGGAATAGGG GTCTGACGCT CAGTGGAACG AAAACTCACG TTAAGGCGCG
CATTGAAGGA GAAGAGAAAA TT

Fig. S2. Antheridium and archegonium development in Δpplas1#26, Δpplas2#16, and Δpplas1Δpplas2#2 plants of *Physcomitrium patens*.

(**a**-**c**) Representative antheridia and archegonia at the gametophore apex in the Δpplas1#26 (**a**), Δpplas2#16 (**b**), and Δpplas1Δpplas2#2 (**c**) plants. "♂" and "♀" indicate the antheridia and archegonium, respectively. Optical sections of an antheridium (**d**) and an archegonium (**e**) in Δpplas1Δpplas2#2 indicate the absence of spermatogenous cells and an egg cell. Scale bars: 50 µm (**a**-**c**) and 20 µm (**d**, **e**). Longitudinal-anticlinal division planes in (**d**) are indicated as "§".

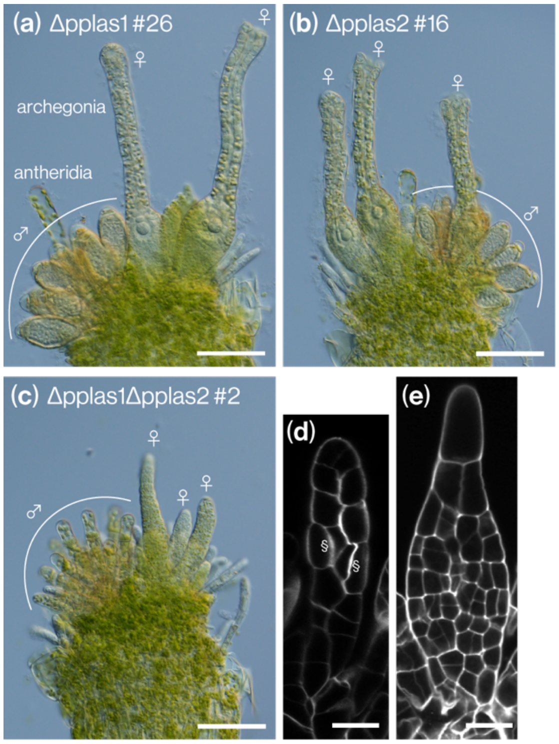

New Phytologist

Fig. S3. Accumulation of PpLAS1 and PpLAS2 fusion proteins during gametangium development in *Physcomitrium patens*.

(**a**-**d**) Representative spatial accumulation patterns of PpLAS1 (**a**, **c**) and PpLAS2 (**b**, **d**) fused to mClover3 fluorescent protein (green) during antheridium (**a**, **b**) and archegonium (**c**, **d**) development in PpLAS1-mClover3#2 and PpLAS2-mClover3#7 plants. Cell walls of antheridia and archegonia were visualized with Calcofluor white (magenta). Arrowheads indicate the antheridium apical stem cell (**a**,**b**) and the archegonium apical stem cell (**c**, **d**) before bilateral cell divisions. White and yellow asterisks indicate apical stem cells and segment cells, respectively. Scale bars: 20 µm.

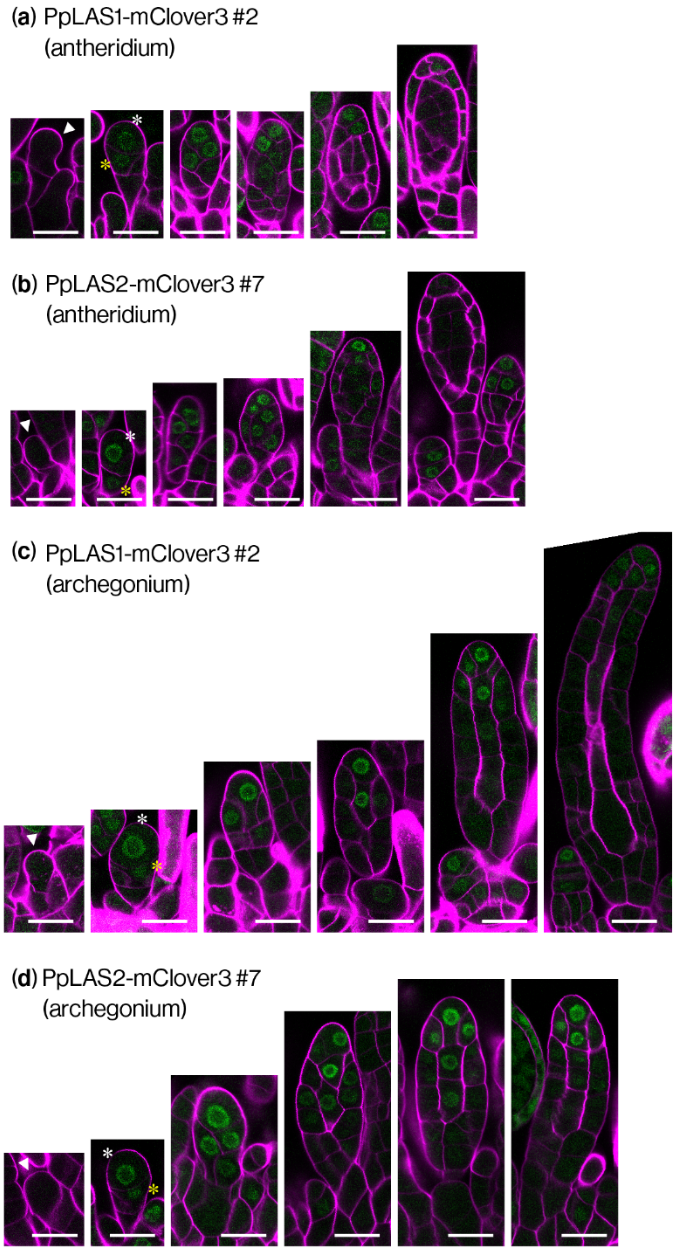

Fig. S4. Formation of gametangia in Δppshr1Δppshr2#7 and nPpLAS1pro:XVE>PpLAS1-CitrineΔpplas1Δpplas2#1 plants of *Physcomitrium patens*.
(**a**) Antheridia and archegonia of Δppshr1Δppshr2#7 plants. (**b**, **c**) Antheridia (**b**) and archegonia (**c**) of nPpLAS1pro:XVE>PpLAS1-CitrineΔpplas1Δpplas2#1 cultivated under the presence of 1 μM β-estradiol for two weeks. Scale bars: 50 μm.

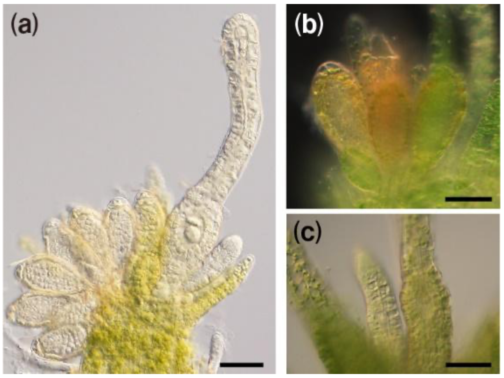

Fig. S5 Effect of cytokinin on antheridia and archegonia development in *Physcomitrium patens*.

(**a-f**) Antheridia (**a-f**) and archegonia (**a, c, e**) of wild-type (**a, b**), Δpplas1Δpplas2#1 (**c, d**), and Δpplas1Δpplas2#2 (**e, f**) plants treated with DMSO (**a, c, e**) or kinetin (**b, d, f**). Gametophores of wild-type and Δpplas1Δpplas2 mutant plants grown on BCD medium under long-day conditions were transferred to BCDAT medium containing either DMSO or 100 µg/L kinetin, and further incubated for three weeks. "♂" and "♀" indicate the antheridia and the archegonium, respectively. Asterisks indicate paraphysis. Scale bars: 100 µm.

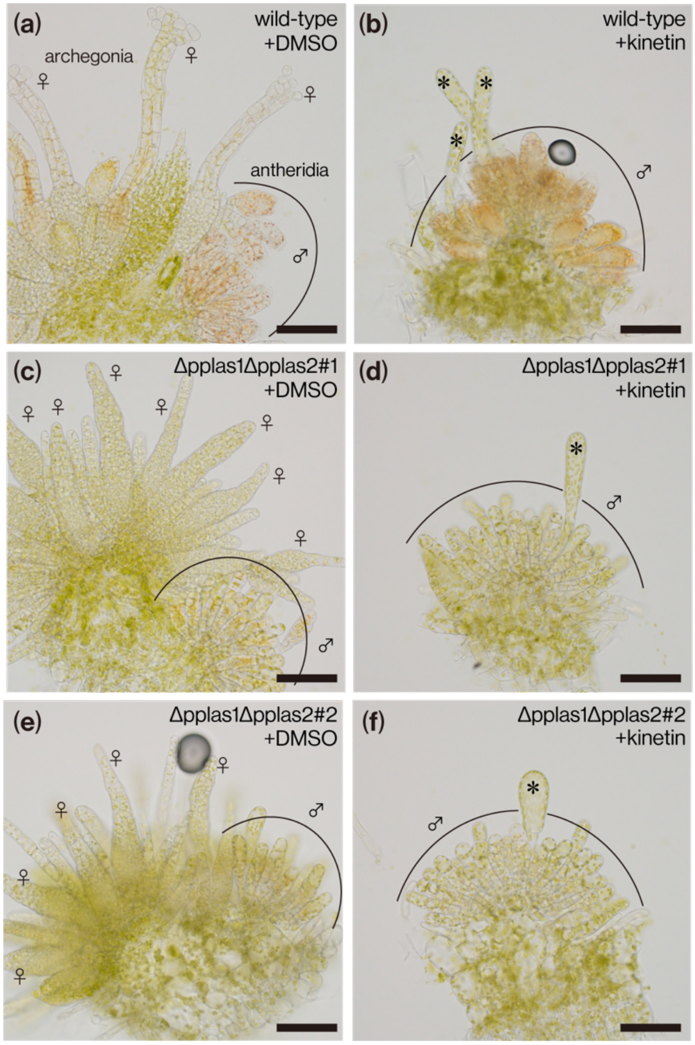

Table S1. Primer sequences used for tail-PCR

Primer name	Sequence (5' -> 3')	Comments
GUS-R3	ATTGACCCACACTTTGCCGTAATGAGTGAC	Use for 1st PCR
GUS-R4	TCTTGTAACGCGCTTTCCCACCAACGCTGA	Use for 2nd PCR
GUSseq-	TCACGGGTTGGGGTTTCTAC	Use for 3rd PCR
A1	NGTCGASWGANAWGAA	
A2	GTNCGASWCANAWGTT	Liu & Whittier, 1995
A3	WGTGNAGWANCANAGA	
